# Understanding Influenza and SARS-CoV-2 Vaccine Hesitancy in Racial and Ethnic Minority Caregivers

**DOI:** 10.3390/vaccines10111968

**Published:** 2022-11-20

**Authors:** Shannon H. Baumer-Mouradian, Rebecca J. Hart, Alexis Visotcky, Raphael Fraser, Swathi Prasad, Michael Levas, Mark Nimmer, David C. Brousseau

**Affiliations:** 1 Department of Pediatrics, Children’s Corporate Center, Medical College of Wisconsin, Milwaukee, WI 53226, USA; 2 Department of Pediatrics, Norton Children’s and University of Louisville School of Medicine, Louisville, KY 40202, USA; 3 Institute for Health and Equity, Division of Biostatistics, Medical College of Wisconsin, Milwaukee, WI 53226, USA

**Keywords:** vaccines/immunizations, influenza, SARS-CoV-2, healthy equity, emergency medicine

## Abstract

(1) Background: We compared influenza and SARS-CoV-2 vaccine hesitancy levels in Black, Hispanic, and White parents/caregivers and identified barriers and facilitators to vaccine acceptance. (2) Methods: This was a mixed methods study. A cross-sectional survey of ED caregivers presenting with children 6mo–18yo compared vaccine hesitancy levels among diverse caregivers. Six focus groups of survey participants, stratified by caregiver race/ethnicity and caregiver intent to receive SARS-CoV-2 vaccine, assessed facilitators and barriers of vaccination, with thematic coding using the Consolidated Framework for Implementation Research (CFIR). (3) Results: Surveys (*n* = 589) revealed Black caregivers had significantly higher vaccine hesitancy rates than White caregivers for pediatric influenza (42% versus 21%) and SARS-CoV-2 (63% versus 36%; both *p* < 0.05). Hispanic caregivers were more hesitant than White caregivers (37% flu and 58% SARS-CoV-2), but this was not significant. Qualitative analysis (*n* = 23 caregivers) identified barriers including vaccine side effects, lack of necessity, inadequate data/science, and distrust. Facilitators included vaccine convenience, fear of illness, and desire to protect others. (4) Conclusions: Minority caregivers reported higher levels of vaccine hesitancy for influenza and SARS-CoV-2. We identified vaccine facilitators and barriers inclusive of Black and Hispanic caregivers, which may guide interventions designed to equitably improve acceptance of pediatric vaccines.

## 1. Introduction

Severe acute respiratory syndrome coronavirus 2 (SARS-CoV-2, which causes the disease known as COVID-19) and influenza viruses place a disproportionately high burden on racial and ethnic minority children. Black and Hispanic children have twice the rate of COVID-19 cases, hospitalizations, and episodes of COVID-19-Associated Multisystem Inflammatory Syndrome compared to White and Asian children [1,2,3]. Similarly, influenza causes doubled hospitalization rates and critical care admissions in Hispanic and Black children, and more than three times higher death rates in Black children compared to White children [4].

While vaccines are shown to reduce the burden of COVID-19 illness as well as reduce hospitalizations and death due to influenza, many children remain unvaccinated. Despite the higher burden of illness in Black and Hispanic children, influenza vaccination rates have historically been lower in Black (B) children compared to White (W) and Hispanic (H) children (49% B, 60% W, 59% H in 2021) [5]. Parent surveys also indicate lower intended SARS-CoV-2 vaccine uptake by Black children (37%) compared to White (47%) and Hispanic (51%) children [6,7].

Vaccine hesitancy (a reluctance or refusal to vaccinate) was named one of the top ten threats to global health by the World Health Organization in 2019 [8]. National surveys indicate that parental vaccine hesitancy toward influenza and SARS-CoV-2 vaccines for children is related to concerns for vaccine side effects, novelty, and perceived lack of effectiveness [7]; however, there is a gap in the literature regarding methods to overcome vaccine hesitancy, specifically in racial and ethnic minority parents. Our study had two goals: (1) to compare levels of vaccine hesitancy for pediatric influenza and SARS-CoV-2 vaccines between Black, Hispanic, and White caregivers, and (2) to identify facilitators and barriers of pediatric vaccine acceptance representative of racial and ethnic minority caregiver thoughts and beliefs. We hypothesized that Black and Hispanic caregivers would have higher rates of vaccine hesitancy compared to White caregivers, and that facilitators and barriers to vaccination would vary based on race/ethnicity.

## 2. Materials and Methods

This is a mixed methods study. Part 1: To measure influenza and SARS-CoV-2 vaccine hesitancy levels and determine factors associated with vaccine hesitancy in Black, White, and Hispanic parents or legal guardians (hereafter referred to as caregivers), we performed a cross-sectional survey of caregivers presenting with their child to the ED. Part 2: To better understand racial and ethnic differences in facilitators and barriers of pediatric influenza and SARS-CoV-2 vaccination, we performed in depth caregiver focus groups by recruiting survey respondents who volunteered to participate.

### 2.1. Part 1: Survey Population and Recruitment

We included English-speaking caregivers of children age 6mo–18yo who presented to a level 1, pediatric emergency department (PED) within an academic children’s hospital in Milwaukee, WI, between February and August 2021. We excluded caregivers of children (1) with an emergent medical condition (emergency severity index (ESI) triage score of ≤2), (2) with concern for abuse or neglect, (3) in law enforcement custody, and (4) admitted. Caregiver receipt of SARS-CoV-2 or the 2020–21 seasonal influenza vaccine was not an inclusion or exclusion criteria. Trained research assistants identified eligible caregivers using the ED trackboard (ESI level), approached participants in person or by phone while in the ED, and enrolled a consecutive sample during pre-determined times, seven days per week. All surveys were completed by caregivers on a research iPad or personal cell phone. If a caregiver presented with more than one child, the survey focused on the youngest vaccine-eligible child.

#### 2.1.1. Survey Data Collection Form Development and Validation

The survey data collection form was derived from the literature (Supplemental Table S1) [9,10]. Tthe primary outcome, caregiver vaccine hesitancy for self was measured by the questions, “How likely are you to receive a flu vaccine this upcoming flu season, September 2021–April 2022?” and “If your doctor felt you were healthy enough and recommended you receive the COVID-19 vaccine, would you get it if it is available?” Caregiver vaccine hesitancy for the child was measured by asking the same questions, replacing “you” with “your child”. To determine factors associated with caregiver vaccine hesitancy for child, we measured caregiver demographic data (including self-reported race and ethnicity), willingness to accept vaccines in the ED, and COVID-19 risk score. COVID-19 risk score included 7 questions on a Likert scale adapted from Malik et al. with higher scores suggesting parental fear of COVID-19 [9]. Study data was managed using REDCap electronic data capture tools [11,12]. To achieve face validity, the survey data collection form was reviewed for health literacy and content by three pediatric emergency medicine faculty and three ED caregivers. Reliability testing was not performed and translation was not required. Our team decided to evaluate vaccine hesitancy for influenza and SARS-CoV-2 vaccines together as both are optional vaccines with poor/expected pediatric uptake to determine if sources of vaccine hesitancy and methods to overcome hesitancy aligned.

#### 2.1.2. Survey Analysis

Vaccine hesitancy/acceptance among Black, Hispanic, and White caregivers was dichotomized, with responses of strongly disagree/disagree/neutral classified as hesitancy and agree/strongly agree indicating acceptance [9]. We calculated that a sample of 584 caregivers would yield 90% power to detect a change in the probability of SARS-CoV-2 vaccine hesitancy from a value of 0.2 at baseline to 0.1, assuming our sample population matched our ED visit population (42% Black, 26% White, and 26% Hispanic), 25% had vaccine hesitancy [9], and that the two-sided test was significant at the 1% level. Descriptive statistics were used to summarize patient and caregiver characteristics. Characteristics were compared using chi-squared tests or Wilcoxon–Mann–Whitney nonparametric tests. Stepwise selection logistic regression models evaluated the effect of race, age, insurance, and COVID-19 risk score for caregiver plans to vaccinate child for influenza and SARS-CoV-2 separately. For this study we conceptualized race and ethnicity as social rather than biological constructs and used previous US Preventative Services Task Force definitions [13].

### 2.2. Part 2: Focus Group Population and Recruitment

Caregiver survey responses from Part 1 were used to stratify caregivers into one of six groups: (1) White (2) Black (3) Hispanic caregivers willing to receive the SARS-CoV-2 vaccine for themselves and (4) White (5) Black (6) Hispanic caregivers unwilling to receive the SARS-CoV-2 vaccine. Following the ED visit, research staff called interested caregivers (identified in the survey) until at least 9 participants committed to attending; focus group attendance was 3–7 caregivers per group.

#### 2.2.1. Focus Group Guide Development, Validation, and Facilitation

Focus group guide questions were adapted from prior studies assessing barriers/facilitators of pediatric influenza vaccine acceptance [14]. To achieve face validity, the focus group questionnaire content and language were reviewed by three pediatric emergency medicine faculty and three ED caregivers. No reliability testing was performed and translation was not required. Focus group questions included caregiver experiences and intentions for themselves and their child regarding influenza and SARS-CoV-2 vaccines (Supplemental Table S2). One-hour focus groups were conducted virtually between June and August 2021. Stipends were mailed to participants at the conclusion of the session. One investigator led the focus groups, and all groups were recorded and transcribed verbatim by a Health Insurance Portability and Accountability Act (HIPAA) compliant transcription service.

#### 2.2.2. Focus Group Thematic Content Analysis

On the basis of the focus group guide and the Consolidated Framework for Implementation Research (CFIR) domain constructs, two investigators developed a provisional codebook of codes and definitions. CFIR is a meta-theoretical framework used in implementation research to evaluate factors affecting implementation through quantitative and qualitative methods [15,16]. After reviewing the first few transcripts, the research team revised the codebook to achieve team consensus on codes and definitions. Each focus group was coded by two investigators and a consensus was reached for any discrepancies. Focus group transcripts were coded deductively based on CFIR constructs and definitions and inductively to identify subthemes within the CFIR domains. CFIR is composed of five domains and was applied accordingly: intervention characteristics (specific characteristics of the vaccine), outer setting (medical/scientific community, government, and media), inner setting (family, friends, and surrounding community), characteristics of individuals (characteristics of the caregiver), and the process of implementation (vaccination process). For the main outcome, we identified the most common barrier and facilitator themes of vaccination for influenza and SARS-CoV-2, using matrices of key themes organized by CFIR domain, representative of at least 5 of 6 focus groups. Similarly, we then identified barrier and facilitator themes of vaccination specifically emphasized by 2 or more Black and/or Hispanic caregivers.

Ethics: This study received Institutional Review Board (IRB) approval from Children’s Hospital of Wisconsin IRB. Survey participants provided consent at the initiation of the survey and consented for the focus groups by volunteering their contact info at the conclusion of the survey.

## 3. Results

### 3.1. Survey

**Figure 1:**
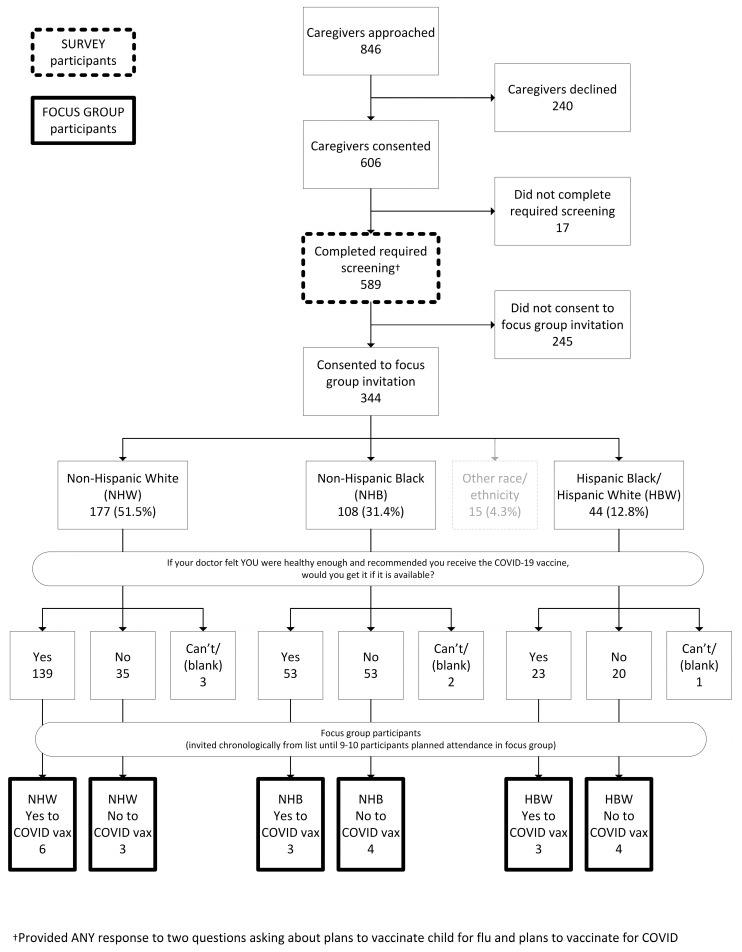
Survey and Focus Group Participant Flow Diagram. This flow diagram illustrates the number of eligible caregivers that initially consented for the survey, those that volunteered to be contacted for focus groups, and those that participated in focus groups.

We approached 846 ED caregivers and 589 (70%) completed the survey. A flow diagram of the study is included (Figure 1). Survey respondents were 47% White, 25% Black, 9% Hispanic, and 9% multi-racial or other. Additional survey participant demographics are listed in Table 1.

**Figure 2:**
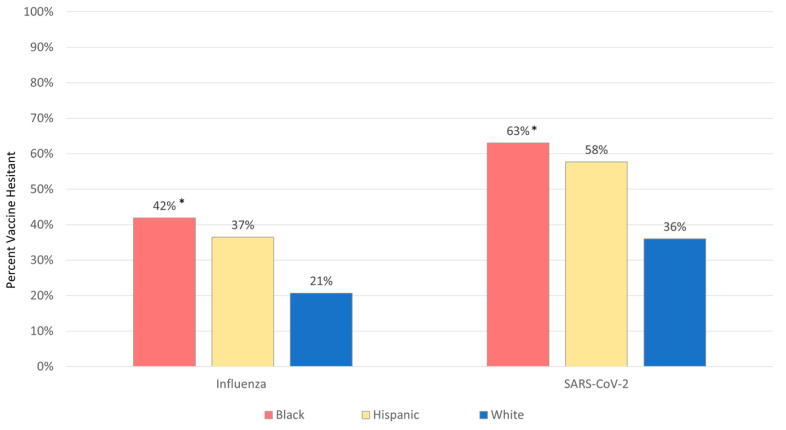
Black Caregivers Report Highest Levels of Vaccine Hesitancy toward Pediatric Influenza and SARS-CoV-2 Vaccines. Pairwise comparisons revealed that Black caregivers were significantly more hesitant to vaccinate children for influenza and SARS-CoV-2 compared to White caregivers. Hispanic caregivers appeared to have greater vaccine hesitancy toward influenza and SARS-CoV-2 than White caregivers, but these did not reach statistical significance. * *p* < 0.05 compared to White.

Thirty two percent (n = 189) of caregivers were hesitant to vaccinate their child for influenza for the 2021–2022 season, and half (50%, n = 295) were hesitant to vaccinate their child for SARS-CoV-2. Black caregivers were significantly more hesitant to vaccinate than White caregivers (influenza 42% vs. 21%, *p* = 0.01, SARS-CoV-2 63% vs. 36%, *p* = 0.01). Hispanic caregivers appeared to have greater vaccine hesitancy than White caregivers, although these differences did not reach statistical significance (influenza 37% vs. 21%, *p* = 0.16 and SARS-CoV-2, 58% vs. 36%, *p* = 0.22) (Figure 2).

A history of caregiver or child influenza vaccine within the past 12mo significantly reduced influenza vaccine hesitancy for the child (*p* < 0.0001 for each). A history of caregiver COVID-19 vaccination, a higher COVID-19 risk score (greater fear), and older caregiver age also reduced COVID-19 vaccine hesitancy for the child (*p* < 0.0001 for each). Logistic regression models demonstrated private insurance was the only significant predictor for caregiver plan to vaccination child for influenza and older age, private insurance, and higher COVID-19 risk score increased the likelihood to plan to vaccinate child for SARS-CoV-2 (Table A1 and Table A2 See Appendix A). Among caregivers planning to vaccinate their child, the pediatric ED was an acceptable location for vaccination among all races/ethnicities, with influenza vaccine acceptance in White 98%, Black 91%, and Hispanic 94% caregivers and SARS-CoV-2 vaccine acceptance in White 96%, Black 86%, and Hispanic 82% caregivers.

### 3.2. Focus Group

Twenty-three survey participants (nine White, seven Black, seven Hispanic) participated in focus groups. Focus group participant demographics are listed in Table A3 (See Appendix A). Two individuals identified as Black and Hispanic but self-selected to participate in the Hispanic focus groups. Fifty eight percent of caregivers had previously been vaccinated for influenza, and fifty four percent had received or intended to receive the SARS-CoV-2 vaccine for themselves (although the majority were already vaccinated).

**Figure 3:**
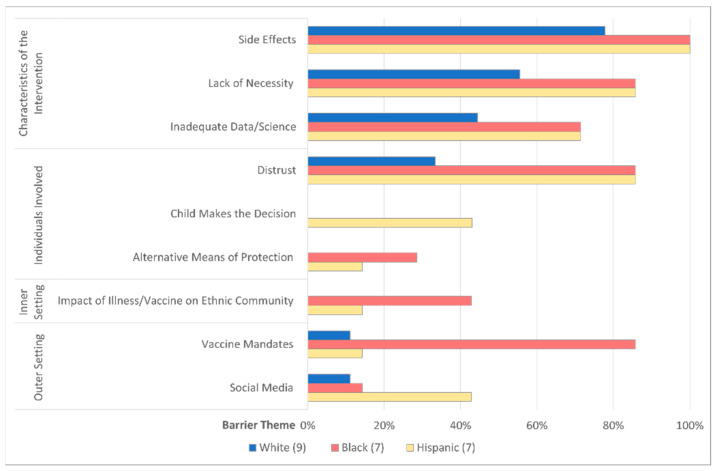
Vaccine Barrier Themes Differ by Race and Ethnicity. Vaccine barrier themes are organized by Consolidated Framework for Implementation Research (CFIR) domains. The bar graph compares the frequency each barrier theme occurred in White, Black, and/or Hispanic participants.

Barriers to influenza and SARS-CoV-2 vaccination centered around four CFIR domains: characteristics of the intervention, individuals involved in the intervention, inner setting, and outer setting. The most common themes noted in at least five of six focus groups included side effects of the vaccine, lack of necessity of the vaccine, inadequate data/science to support the vaccine, and distrust of the medical system/science/government. Table A4 (See Appendix A) defines all barrier themes by CFIR domain, provides an example quote, and outlines the distribution of each theme across the various focus groups. Figure 3 demonstrates the distribution of the barrier themes sub-grouped by race and ethnicity. Black and Hispanic caregivers specifically emphasized distrust, impact of vaccination on the community, resistance toward vaccine mandates, and social media as important barriers. Hispanic caregivers uniquely described the child making the decision (not) to vaccinate as a potential barrier.

Facilitators of both influenza and SARS-CoV-2 vaccination were organized around four CFIR domains including implementation process, individuals involved in the intervention, inner setting, and outer setting. The most common themes noted in at least five of six focus groups included convenience, provider messaging, mental and physical preparation, fear of illness, desire to protect others, vaccine beliefs of family, friends, and community, and doctor’s recommendation. Table A5 (See Appendix A) defines all facilitator themes, provides example quotes, and outlines the distribution of each theme across the various focus groups.

**Figure 4:**
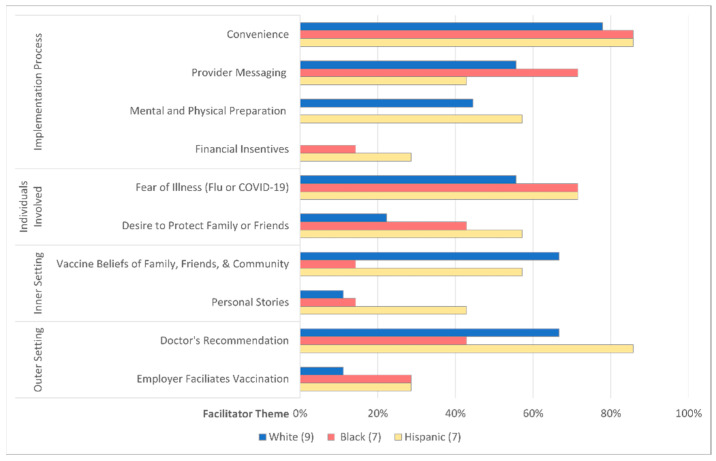
Vaccine Facilitator Themes Differ by Race and Ethnicity. Vaccine facilitator themes are organized by Consolidated Framework for Implementation Research (CFIR) domains. The bar graph compares the frequency each facilitator theme occurred in White, Black, and/or Hispanic participants.

Figure 4 demonstrates the distribution of the facilitator themes sub-grouped by race and ethnicity.

Convenience, fear of illness, and desire to protect family, friends, and community were significant facilitators across all races and ethnicities. Among caregivers identifying as Hispanic or White, mental and physical preparation, vaccine beliefs of family, friends, and community, and a doctor’s recommendation were common facilitators. In contrast to simply receiving a doctor’s recommendation, Black caregivers highlighted provider messaging, the concept of communicating about vaccines in a non-judgmental, transparent way as a crucial method to promote vaccines, and this facilitator was also echoed by other White and Hispanic caregivers. Additionally, Hispanic and Black caregivers emphasized themes such as a desire to protect others, employer facilitation of vaccination, and personal stories from others as facilitators.

ED vaccinations were reported to be a convenient option, and thus were seen as a facilitator across all six focus groups. Barriers to ED vaccinations included a lack of mental and physical preparation time and a concern for difficulty scheduling a second dose of SARS-CoV-2 vaccine. (This study occurred prior to Centers for Disease Control and Prevention’s recommendation for a 3rd dose of SARS-CoV-2 vaccine for children.)

## 4. Discussion

In summary, we confirmed that Black caregivers reported higher vaccine hesitancy toward both influenza and SARS-CoV-2 vaccines for children compared to White caregivers. Factors associated with lower vaccine hesitancy were consistent with prior literature and included prior influenza vaccination (for caregiver and/or child within 12 mo), prior caregiver intent to vaccinate self for SARS-CoV-2, as well as a fear of COVID-19 (higher COVID risk score) [7,9,17,18]. Qualitative analysis across all races/ethnicities demonstrated side effects, lack of necessity, perceived inadequate data/science, and distrust as barriers to vaccination; alternatively, convenience in obtaining/scheduling a vaccine, fear of illness, and desire to protect others were the primary vaccine facilitators.

Our study is novel in that we call attention to Black and Hispanic caregiver voices and experiences, and we propose potential solutions drawn from the suggestions made in our focus groups. Black and Hispanic caregivers specifically emphasized distrust of the medical system/science/government, impact of vaccination on the community, resistance toward vaccine mandates, and social media as important barriers to vaccination. Among Black participants, facilitators centered around provider messaging and employer facilitation of vaccination; therefore, interventions may include: (1) scripting provider messaging to promote a discussion about the risks and benefits of vaccines in a neutral, non-judgmental tone, (2) providing caregivers paid time off to get the child vaccinated, and (3) utilizing community messaging to share the impact of disease and the specific benefits of the vaccine to the Black community. In contrast, Hispanic participants emphasized personal stories, financial incentives, and youth as the decision-maker as facilitators. Subsequently, possible interventions may include: (1) having community vaccination sites and/or community leaders endorsing vaccinations, (2) publicizing personal stories regarding successful vaccinations in the Hispanic community, (3) targeting vaccine promotional strategies to youth and (4) providing financial incentives for vaccination.

Prior studies have noted the importance of a strong doctor’s recommendation to promote vaccination [7,19]. Our study uniquely adds that many caregivers, especially Black caregivers, were more likely to be influenced by the provider approach rather than a simple recommendation. These caregivers emphasized the desire for provider messaging to be presented in a neutral tone, discussing benefits as well as risks of the vaccine, admitting limitations in provider knowledge, and empowering caregivers to make the decision to vaccinate their child. This theme is supported by prior work in motivational interviewing, necessitating the need for a respectful empathetic discussion about vaccines to build a strong relationship between the caregiver and provider [20,21,22,23,24].

Unfortunately, provider time and resources are often limited, and it may not be feasible to individualize vaccine messages to each diverse patient/caregiver. Therefore, universal strategies that equitably promote pediatric influenza and SARS-CoV-2 vaccines may be a more effective way to address vaccine disparities. Combining a doctor’s recommendation with scripted provider messaging is one potential method that may bridge these needs. Additionally, providing vaccination in non-traditional settings such as the ED may also increase equitable access and uptake. Prior work by the authors and others has demonstrated that the ED provides a unique opportunity to overcome disparities in pediatric vaccination, as the ED frequently serves those without a primary care provider, overcomes access barriers, and provides additional opportunities to identify and overcome vaccine hesitancy [25,26,27,28]. In this study, we identified that the majority of caregivers intending to vaccinate their child found the ED an acceptable location to receive pediatric influenza and SARS-CoV-2 vaccines, regardless of caregiver race and ethnicity. Additionally, convenience was the most universal facilitator of vaccination noted in focus groups, with participants from each focus group identifying the ED as a convenient location for pediatric vaccination.

Addressing vaccine hesitancy also requires public health officials to address vaccine barriers. The barriers identified in this study are consistent with prior studies where pediatric caregivers [10,29,30,31,32], as well as Black and Hispanic adults [24,32,33], identified safety, side effects, misinformation, and mistrust of the medical system as barriers to influenza and SARS-CoV-2 vaccination. Given distrust was reported commonly in Black and Hispanic caregivers, efforts to restore the relationship between the medical community and minoritized communities are needed. Our focus group participants suggested interventions such as (1) bringing providers into schools to offer vaccines, (2) hosting community nights to address caregiver vaccine questions, or (3) going door to door to offer vaccines, to help establish a trusting presence in the community.

Additionally, most Black and Hispanic caregivers were resistant to pediatric vaccine mandates for schools and daycares, highlighting the importance of the caregiver’s right to choose. Some caregivers even reported they would remove their child from school if vaccines became required, especially for SARS-CoV-2 vaccines. These findings are consistent with our recent multi-center international survey of 21 pediatric EDs across four countries, which confirmed that many parents were resistant to SARS-CoV-2 vaccine mandates in schools [34].

Finally, our findings suggest that parent acceptance of influenza and SARS-CoV-2 may ultimately depend on more longitudinal safety and efficacy data. Future research is necessary to understand the effects of the emergency use authorization process on parent mistrust, particularly in minoritized communities.

## 5. Limitations

This study was conducted in a single tertiary care ED and may not be generalizable to the total population, especially given the majority of patients presented for low acuity complaints and over half had public insurance or lack of insurance. The survey was conducted as a convenience sample and all answers were anonymous; therefore, the research team was unable to confirm if child COVID-19 vaccination occurred at a later time. Additionally, the race/ethnicity caregiver mix may not be nationally representative, and small sample size within the focus groups may not be adequate to sufficiently capture all emergent or dominant themes. Finally, selection bias may exist as the survey population overrepresented the Non-Hispanic White population in our ED and underrepresented the Non-Hispanic Black and Hispanic White/Hispanic Black populations.

Due to limited personnel, the race and ethnicity of the focus group facilitator did not match the Black and Hispanic focus group participants; therefore, participants may have been more reserved with their comments. Additionally, Spanish translation and interpreters were not available for the survey or focus groups; therefore, selection bias may have been introduced, as we were unable to recruit Spanish-speaking caregivers. Notably, two individuals in the Hispanic focus groups also identified as Black; these caregivers chose to participate in the Hispanic focus groups, but responses may not adequately capture the intersection of these identities. While we stratified focus groups based on caregiver history/intent of SARS-CoV-2 vaccination to ascertain opinions based on likelihood of vaccine acceptance, this history did not consistently predict caregiver opinions toward influenza or SARS-CoV-2 vaccines for self and child. Additionally, we were unable to confirm whether or not the caregivers and or children went on to receive either vaccination.

## 6. Conclusions

Black caregivers report higher rates of influenza and SARS-CoV-2 vaccine hesitancy compared to White caregivers. Our focus groups highlighted Black and Hispanic caregiver voices and identified pediatric vaccination barriers and facilitators inclusive of these minority caregivers. We propose interventions based on focus group themes that may promote pediatric influenza and SARS-CoV-2 vaccines among Black and Hispanic caregivers, highlighting two strategies that may universally promote equitable vaccine uptake: (1) combining a strong doctor’s recommendation with scripted provider messaging and (2) promoting vaccines in the ED. Further studies to design and implement such interventions are needed to determine their ultimate effect on vaccine uptake among minority populations.

## Figures and Tables

**Table 1:** Survey Participant Demographics.

Variables	Total n = 589 (col %)
**Parent Gender**	
Male	101 (17.1)
Female	468 (79.5)
Other	2 (0.3)
Missing	18 (3.1)
**Parent Age**	
18–25	60 (10.2)
26–35	209 (35.5)
36–45	222 (37.7)
46+	81 (13.8)
Missing	17 (2.8)
**Parent Education**	
No high school	15 (2.6)
Some high school/High school diploma/GED	138 (23.4)
Some college/College degree	277 (47.0)
Graduate/Professional degree	141 (23.9)
Missing	18 (3.1)
**Parent Race and Ethnicity**	
Non-Hispanic White	277 (47.0)
Non-Hispanic Black	148 (25.1)
Hispanic Black or Hispanic White	52 (8.8)
More than one race, regardless of Ethnicity	26 (4.4)
Other race	18 (3.1)
Missing	68 (11.6)
**Parent Insurance**	
Private or Commercial Insurance	293 (49.8)
Public Insurance (CHIP, Badger care, Medicaid, Medicare)	268 (45.5)
No insurance or Unsure	9 (1.5)
Missing	19 (3.2)

The survey was conducted between February and August 2021. The majority of survey participants were female, age 26–45yo, with some college or higher education. 47% White, 25% Black, and 9% Hispanic. 50% had private insurance.

## Data Availability

Not applicable.

## Supplementary Materials

The following supporting information can be downloaded at: https://www.mdpi.com/article/10.3390/vaccines10111968/s1, Table S1: Caregiver Survey Questions; Table S2: Focus Group Facilitator Guide.

## Appendix A

**Table A1:** Logistic Regression Model for Plans to Vaccinate Child for Influenza.

Effect	*Estimate*	*95% Confidence Limits*		*p-Value*
**Race**				0.0566
Hispanic Black or White vs. Non-Hispanic White	0.649	0.329	1.280	0.2125
More than one race, regardless of Ethnicity vs. Non-Hispanic White	0.319	0.132	0.771	0.0111
Non-Hispanic Black vs. Non-Hispanic White	0.547	0.332	0.902	0.0181
Other race vs. Non-Hispanic White	0.647	0.223	1.880	0.4240
**Insurance**				0.0024
No insurance or Unsure vs. Private or Commercial Insurance	0.509	0.106	2.444	0.3986
Public Insurance (CHIP, Badger care, Medicaid, Medicare) vs. Private or Commercial Insurance	0.446	0.283	0.704	0.0005

Logistic regression models demonstrated private insurance was the only significant predictor for caregiver plan to vaccination child for influenza.

**Table A2:** Logistic Regression Model for Plans to Vaccinate Child for SARS-CoV-2.

Effect	*Odds Ratio*	*95% Confidence Limits*		*p-Value*
**Race**				0.0529
Hispanic Black or White vs. Non-Hispanic White	0.633	0.307	1.304	0.2150
More than one race, regardless of Ethnicity vs. Non-Hispanic White	0.833	0.294	2.355	0.7302
Non-Hispanic Black vs. Non-Hispanic White	0.513	0.301	0.874	0.0141
Other race vs. Non-Hispanic White	2.049	0.652	6.442	0.2195
**Age**				0.0002
18–25 vs. 46+	0.219	0.089	0.543	0.0010
26–35 vs. 46+	0.240	0.120	0.481	<0.0001
36–45 vs. 46+	0.452	0.228	0.894	0.0226
**Insurance**				0.0176
No insurance or Unsure vs. Private or Commercial Insurance	0.255	0.035	1.864	0.1782
Public Insurance (CHIP, Badger care, Medicaid, Medicare) vs. Private or Commercial Insurance	0.511	0.313	0.833	0.0071
**COVID Risk**	1.697	1.468	1.962	<0.0001

Logistic regression models demonstrated older age, private insurance, and higher COVID-19 risk score increased the likelihood to plan to vaccinate child for SARS-CoV-2.

**Table A3:** Focus Group Participant Demographics.

Focus Group	Parent ID	Race/ Ethnicity	Parent Intent to Receive COVID-19	Prior Parent Influenza	Intent to Vaccinate Child for Flu	Intent to Vaccinate Child for COVID-19	Gender	Age	Education	Insurance
1	1	Non-Hispanic White	Definitely Yes	Yes	Definitely Yes	Definitely Yes	Female	46–55	Graduate/ Professional degree	Private/ Commercial
	2							36–45		
	3								Some college/ College degree	
	4					Probably Yes	Male	55+		
	5		Probably Yes		Probably Yes			26–35		
	6			No		Definitely no	Female	36–45		
2	7	Non-Hispanic White	Definitely no	Yes	Probably yes	Definitely no	Female	36–45	Some college/ College degree	Public
	8			No				26–35		Private/ Commercial
	9			Yes			Male			
3	10	Non-Hispanic Black	Definitely yes	Yes	Probably yes	Unsure	Female	36–45	Some college/ College degree	Private/Commercial
	11		Probably yes		Definitely yes	Definitely yes		26–35	Some high school	Public
	12				Probably no	Probably no			Some college/ College degree	Private/Commercial
4	13	Non-Hispanic Black	Unsure	Yes	Probably yes	Unsure	Female	26–35	No high school	Public
	14		Definitely no	No		Definitely no		18–25	Some college/ College degree	
	15				Definitely no			36–45		
	16							26–35	Some high school	
5	17	Hispanic White	Definitely yes	Yes	Definitely yes	Definitely yes	Female	18–25	Some high school	Public
	18			No	Probably no	Probably yes		36–45	Graduate/ Professional degree	Private/Commercial
	19	Hispanic White/Black	*Not* *answered*	Yes	Probably yes	Probably no		26–35	Some college/ College degree	Public
6	20	Hispanic Black	Unsure	No	Unsure	Probably no	Female	26–35	Some high school	Public
	21	Hispanic White	Definitely no		Probably no	Definitely no			Some college/ College degree	
	22		Definitely yes		Probably yes	Definitely yes		36–45	Some high school	Private/Commercial
	23		Unsure	Yes	Unsure	Unsure		26–35	Some college/ College degree	Public

The focus groups were conducted between June and August 2021. Caregivers focus group number, influenza and SARS-CoV-2 vaccine status, intent to vaccinate child for influenza and SARS-CoV-2 and additional demographics are included. 58% of participants had previously received influenza vaccine and 54% previously had or intended to receive SARS-CoV-2 vaccine for self.

**Table A4:** Barrier Themes to Influenza and SARS-CoV-2 Vaccination.

CFIR Domain/Theme	Description of Facilitator	Supporting Quote	Distribution of Themes
**Characteristics of the Intervention**			
Side Effects	Concern about prior vaccine tolerance including short-term and long-term side effects of vaccines was expressed.	“I find it really concerning…the amount of adverse reactions that are reported in VAERS are like disproportionate to flu vaccine… there’s enough young men that have had heart issues related to it, seems like a very real concern for me to have a couple of young boys. I don’t know how it’s going to affect them. It’s very unproven technology.” (W, anti)	6FG, (2W, 2B, 2H)
Lack of Necessity	A perception that the vaccine is unnecessary due to a lack of vaccine effectiveness at preventing illness, a low perceived severity of influenza or COVID-19 illness, or a low risk for illness due to low risk past medical history, occupation, or home/school environment.	“I don’t think it’s important…to get the children vaccinated with the COVID vaccine right now… Maybe if we had more evidence that children are getting COVID like adults were getting COVID, but adults were dropping dead from COVID, children are not. (B, pro)	6 FG, (2W, 2B, 2H)
Inadequate Data/ Science	The lack of available information regarding the facts behind vaccine development including length of time and number of people in whom vaccines have been tested. Vaccine novelty, rushed development process, and a lack of adequate follow-up time to assess for side effects were also included in this theme.	“I’m not getting my kids vaccinated because this vaccination shot came about so fast. It almost takes about 10 years to make an actual vaccination shot, so how did they make one in nine months? You’re not giving us enough information, y’all just want us to get this shot and be okay with that.” (B, anti)	6 FG, (2W, 2B, 2H)
**Individuals Involved in the Intervention**			
Distrust	Caregiver distrust that vaccines have been developed in a safe and efficacious manner. Specifically, concerns were raised that children should not be treated as “test subjects”, the science used to develop the vaccine was not sound, the provider recommending the vaccine was withholding information or lacks sufficient knowledge of vaccine, or that CDC and US government were promoting vaccines for personal gain or other nefarious reasons.	“I don’t know what goes into the vaccine, but…I’ve looked into it and it’s a bunch of words I can’t pronounce. …I’ll be honest…I don’t really necessarily trust that, and you know, right now pharmaceutical companies (don’t) have the people’s best interests at heart…I feel like it’s more of a money thing than keeping the public safe.” (H, anti)	5 FG, (1W, 2B, 2H)
Child Makes the Decision	Most notably in Hispanics, parent deferred to the child to decide whether to obtain the vaccine.	“I really leave it up to my 16, almost 17yo. I left it up to her whether she wanted to take it, and she refused and she didn’t want to because she was scared of the things that she’s seen on the news.” (H, anti)	2 FG (2H)
Alternative Means of Protection	Some families utilized social distancing, masking, and hand hygiene to protect against illness and believed vaccines were not necessary.	“I’m still on the fence about it because…I feel healthy. I practice, you know, the safe guidelines of masking up when it’s required and necessary. So I’m still not convinced that I should, or that I even want to [get the shot].” (H, anti)	4 FG (2B, 2H)
**Inner Setting**			
Impact of Illness/ Vaccine on Ethnic Community	Most notably in Blacks and Hispanics, the effects of influenza or COVID-19 illness or vaccinations on their specific communities, as well as concerns about how their community has historically been treated or impacted by the medical community.	“We’re the community that’s being affected the most by COVID. The people are dying the most by COVID. We’re not the ones trying to jump in the forefront and be those people that could potentially end up getting killed by this vaccine.” (B, pro)	3 FG (2B, 1H)
**Outer Setting**			
Vaccine Mandates	Requirements to obtain vaccine for school, employment, or other reasons as well as the right of the parent to choose if child should be vaccinated. Most parents believe parent should have the right to determine if child got vaccinated, but many reported they would only get the vaccine if mandated.	“If people have a choice, they’re more comfortable with choosing your own, versus being forced. I think that’s why a lot of people haven’t got the COVID vaccine ‘cause they’re not comfortable because they feel like, oh they want to force me or change me or make me do something I don’t want to do.” (B, pro)	4 FG (1W, 2B, 1H)
Social media	Social media and news portrayal of vaccines.	“You hear all these horrific stories about people that have had the vaccine and they end up dying right after the vaccine. And all the news doesn’t help the situation, you know, persuade people that are trying to get vaccinated. It’s all about the media, too…it scares people.” (H, anti)	4 FG (1W, 1B, 2H)

Barrier themes are organized by Consolidated Framework for Implementation Research (CFIR) domains. Each theme is accompanied by a description, supporting quote, and distribution of theme appearance within the focus groups. The quote author is identified by the participant’s race (W-White, B-Black, H-Hispanic) and SARS-CoV-2 vaccine status (pro- intended to receive for self, anti- doesn’t intend to receive). Distribution of themes column includes the number of focus groups (FG) where the theme appeared and the race/ethnicity of the participants in these FGs.

**Table A5:** Facilitator Themes to Influenza and SARS-CoV-2 Vaccination.

CFIR Domain/Theme	Description of Facilitator	Supporting Quote	Distribution of Theme
**Implementation Process**			
Convenience	Ease of obtaining a vaccine, via providing vaccines at common locations, a variety of flexible appointment times around school and work schedules and facilitating dose 2 scheduling for SARS-CoV-2.	“I think when they rolled out, for COVID vaccine, they made it so convenient for people to get vaccinated. I didn’t find it hard to get vaccinated for COVID at all as an adult. Maybe if…the time [comes] and it’s acceptable for children to get it, at the doctor’s office, make it as easy for us to get our kids vaccinated as you did adults.” (B, pro)	6 FG, (2W, 2B, 2H)
Provider Messaging	How and what providers communicate to families to promote vaccines. Parents prefer a neutral tone, that is sensitive to the parent/child’s culture and avoids pro-vaccine bias. Parents desire transparent messaging with data on the benefits, side effects, and long-term risks of vaccines.	“If they can explain to me more about, like, what’s in these shots. Like, what’s the medicine, what’s the outcome of the medicine. …And not just the good, I [want to] hear the bad too.” (B, anti)	5 FG, (2W, 2B, 1H)
Mental and Physical Preparation	The process of preparing for vaccination, this includes mentally reviewing vaccine information, making a decision prior to being offered, and emotionally preparing the child for vaccination. Physically, parents prepared supplies (acetaminophen and electrolyte fluids) to address side effects and scheduled time off from work.	“Now we mentally prepare and we’re like, “Okay, we’re going to the pediatrician, we’re getting these shots. Let’s all take a deep breath. Like let’s get takeout for dinner, right? Like we plan to take care of ourselves. And one of our kiddos is prone to getting like a spiking of fever. So, we…know that now and we’re ready with the medicines.”(W, pro)	5FG, (2W, 1B, 2H)
Financial Incentives	Offering stipends or gift cards to receive vaccine or making vaccines free of charge.	“I feel like if they were to get, like, 10% off Target gift card if they got their flu shot at CVS pharmacy or whatnot, like, I feel like that would kind of…help persuade people.” (H, anti)	2 FG (1B, 1H)
**Individuals Involved in the Intervention**			
Fear of Illness (Flu or COVID-19)	A concern about becoming ill from virus as well as a desire to be protected from illness.	“…It’s just been a really, really crazy high number of deaths with it. So that scares all of us and…that puts perspective that you might have a better chance of life with the vaccine.” (H, pro)	6 FG, (2W, 2B, 2H)
Desire to Protect Family, Friends, & Community	Desire to obtain vaccine to prevent illness transmission to high-risk family members, friends, or other community members.	“I got it [vaccine] for myself because my parents are older, and they were completely terrified. We spent the past year and a half away from them. …They would not let us in the house, and we were a very close family, so that was extremely hard. So, I kind of balanced it out—like, let me take this risk for them. They got the vaccine. I’ll take the vaccine for them.” (W, anti)	6FG, (2W, 2B, 2H)
**Inner Setting**			
Vaccine Beliefs of Family, Friends, & Community	The impact of family, friends, or the local community’s vaccination status & vaccine beliefs on the caregiver’s decision to vaccinate.	“Everybody in my extended family is vaccinated. My sister …she’s a social worker in a dialysis clinic…And so she was like the first in line to get vaccinated along with other healthcare workers. So yeah, we’re all vaccinated. I would definitely plan to vaccinate the kiddos once they’re able to be.” (W, pro)	5FG, (2W, 1B, 2H)
Personal Stories	Personal experiences of self or others with illness or vaccine that affected caregiver decision-making around vaccines.	“When my nephew was born, he was a month old and he had Influenza B, and…we almost lost him. So, I’m very pro getting the flu shot.” (H, anti)	4 FG (1W, 1B, 2H)
**Outer Setting**			
Doctor’s Recommendation	A positive recommendation for vaccination from the child’s primary physician.	“We always trust our doctor’s suggestions. So even like, you know, with the COVID vaccine for myself and my two older kids who are eligible…if my doctor recommends it versus if she doesn’t, that’s our big decision maker.” (W, pro)	5FG, (2W, 1B, 2H)
Employer Facilitates Vaccination	Employers provides caregivers time off or compensation for getting their child vaccinated.	“Jobs provide time for parents to take off to go get their children vaccinated and you pay them for it.” B, pro)	4FG (1W, 1B, 2H)

Facilitator themes are organized by Consolidated Framework for Implementation Research (CFIR) domains. Each theme is accompanied by a description, supporting quote, and distribution of theme appearance within the focus groups. The quote author is identified by the participant’s race (W-White, B-Black, H-Hispanic) and SARS-CoV-2 vaccine status (pro- intended to receive for self, anti- doesn’t intend to receive). Distribution of themes column includes the number of focus groups (FG) where the theme appeared and the race/ethnicity of the participants in these FGs.
