# Supplementary material for: Understanding Influenza and SARS-CoV-2 Vaccine Hesitancy in Racial and Ethnic Minority Caregivers
Source: Vaccines (Basel). 2022 Nov 20;10(11):1968. doi: 10.3390/vaccines10111968 (PMC9697963; doi:10.3390/vaccines10111968)
Supplement: Supplementary file 1 [file vaccines-10-01968-s001.zip › vaccines-1945826-supplementary.pdf]

Table S1. Survey Questions.

**1**	**Have you had a flu vaccine during the last 12months? The flu vaccine could have been a shot or a nose spray.**
2	How likely are **you** to receive a flu vaccine this upcoming flu season, September 2021-April 2022?
3	Has **your child** had a flu vaccine during the last 12 months?
4	In the past, where has **your child** received his or her flu shot?
5	How likely is **your child** to receive a flu vaccine this upcoming flu season, September 2021-April 2022?
6	There are many reasons why people don't get flu vaccines. What is the main reason that **your child** will probably not/definitely not/unsure if get a flu vaccine this season?
7	If your child's doctor felt **your child** was healthy enough to get the flu vaccine while in the emergency department, would **your child** get it?
8	Have **you, your child, or someone that lives with you** had COVID-19 symptoms or been diagnosed with COVID-19?
9	Has any family member or close friend you know died due to COVID-19?
10	If your doctor felt **you** were healthy enough and recommended you receive the COVID-19 vaccine, would **you** get it if it is available?
11	If your child's doctor felt **your child** was healthy enough and recommended the COVID-19 vaccine, would **your child** get the vaccine if it is available?
12	There are many reasons why people don't get vaccines. What is the main reason that **your child** will probably not/definitely not/unsure if get a COVID-19 vaccine?
13	If your child's doctor felt **your child** was healthy enough to get the COVID-19 vaccine while in the emergency department, would **your child** get vaccinated?
14	Please choose how much you agree with this statement: My child's health will be severely damaged if he or she gets COVID-19.
15	Please choose how much you agree with this statement: I think COVID-19 is more severe than flu.
16	Please choose how much you agree with this statement: If my child gets sick with something else or gets injured, we prefer not to go to a hospital because of the risk of getting COVID-19 in the hospital.
17	Please choose how much you agree with this statement: COVID-19 will cause serious damage in my community.
18	Please choose how much you agree with this statement: COVID-19 will spread widely in the United States.
19	Please choose how much you agree with this statement: My child is more likely to get COVID-19 than other people.
20	Please choose how much you agree with this statement: I believe I can protect my child against COVID-19 better than other people.

21	**Your** Gender
22	**Your** Age
23	**Your** Education
24	**Your** Race
25	**Your** Ethnicity
26	**Your** type of Insurance
27	**Your** zip code
28	Following this survey, would you be willing to be contacted by our research staff to participate in a virtual focus group to discuss your opinions on influenza and COVID-19 vaccines? You will get a $100 VISA gift card if you choose to participate.
29	In order for us to contact you to participate in a focus group, please provide the following information: **Your first and last name**.
30	Please provide your phone number.
31	Please provide your email.
32	What address can we use to mail your gift card if you complete a focus group?

Table S2. Focus Group Questions.

Item	Flu vaccine interview questions
1	Flu Shots/flu vaccines are available every year. Tell me about your child getting the flu shot. What do you think about your child getting the flu shot every year?
2	Tell me about how important you think it is for your child to get a flu shot every year. Why do you think it is important or not important?
3	Tell me about your thoughts about why you don't have your child get the flu shot every year.
4	Was there anything that makes it hard to get your child a flu shot?
5	What would help you make a decision about your child getting the flu shot?
6	Is there a person that could help you make the decision about your child getting a flu shot?
7	If you had (… info) or if (…person) helped you make a decision, what would change about getting your child a flu shot?
8	If you had (… info) or if (…person) helped you make a decision, would that inspire your friends or family to make a decision about getting a flu shot for their child?
9	What if your doctor recommended the flu shot each year, how would that impact your decision to get your child vaccinated?

10	If I told you 113 children die each year from influenza and … are hospitalized, how would that impact your decision to get your child vaccinated?
11	What would make it easier for you to get your child the flu shot each year?
12	Tell me about what doctors, hospitals, leaders in the community, or the government could do to inspire you, your friends, and your family to get the flu shot?
13	If your child’s doctor felt your child was healthy enough to get the flu shot while in the emergency room, tell me about whether you would have your child get the flu shot or not. Tell me about how you decided this.
14	If you could get the flu shot for yourself in the emergency room, tell me about whether you would get the flu shot. Tell me about if you would consider having your other children get the flu shot in the emergency room even if they weren’t patients.
	**COVID-19 Vaccine interview questions**
1	Tell me about whether your friends and families will get a COVID-19 vaccine, when available. Tell me more about how your friends and family might decide this.
2	Tell me about how important you think it is for your child to get the COVID-19 vaccine, when available. Why do you think it is important or not important?
3	Is there anything that will make it hard to get your child a COVID-19 vaccine?
4	What would help you make a decision about your child getting the COVID-19 vaccine?
5	Is there a person that could help you make the decision about your child getting the COVID-19 vaccine?
6	If you had (… info) or if (…person) helped you make a decision, what would change your mind about getting your child a COVID-19 vaccine?
7	If you had (… info) or if (… person) helped you make a decision, would that inspire your friends or family to make a decision about getting a COVID-19 vaccine for their child?
8	What if your doctor recommended the COVID-19 vaccine for your child, how would that impact your decision to get your child vaccinated?
9	If I told you within the first three months of COVID-19 in the US, 2,500 children were infected and 3 children died, how would that impact your decision to get your child vaccinated?
10	What would make it easier for you to get your child the COVID-19 vaccine?
11	Tell me about what doctors, hospitals, leaders in the community, or the government could do to inspire you, your friends, and your family to get the COVID vaccine?
12	If your child’s doctor felt your child was healthy enough to get the COVID-19 vaccine while in the emergency room, tell me about whether you would have your child get the COVID-19 vaccine or not. Tell me about how you decided this.

13	If you could get the COVID-19 vaccine for yourself in the emergency room, tell me about whether or not you would get it. Tell me about if you would consider having your other children get the COVID-19 vaccine in the emergency room even if they weren’t patients.
